# Gaming Against Frailty: Effects of Virtual Reality-Based Training on Postural Control, Mobility, and Fear of Falling Among Frail Older Adults

**DOI:** 10.3390/jcm14155531

**Published:** 2025-08-06

**Authors:** Hammad S. Alhasan, Mansour Abdullah Alshehri

**Affiliations:** Department of Medical Rehabilitation Sciences, Faculty of Applied Medical Sciences, Umm Al-Qura University, Mecca 24382, Saudi Arabia

**Keywords:** frailty, virtual reality, exergaming, postural control, balance confidence, Nintendo Ring Fit Plus™, older adults, falls, physiotherapy, functional mobility

## Abstract

**Background/Objectives:** Frailty is a prevalent geriatric syndrome associated with impaired postural control and elevated fall risk. Although conventional exercise is a core strategy for frailty management, adherence remains limited. Virtual reality (VR)-based interventions have emerged as potentially engaging alternatives, but their effects on objective postural control and task-specific confidence in frail populations remain understudied. This study aimed to evaluate the effectiveness of a supervised VR training program using the Nintendo Ring Fit Plus™ on postural control, functional mobility, and balance confidence among frail community-dwelling older adults. **Methods:** Fifty-one adults aged ≥65 years classified as frail or prefrail were enrolled in a four-week trial. Participants were assigned to either a VR intervention group (*n* = 28) or control group (*n* = 23). Participants were non-randomly assigned based on availability and preference. Outcome measures were collected at baseline and post-intervention. Primary outcomes included center of pressure (CoP) metrics—sway area, mean velocity, and sway path. Secondary outcomes were the Timed Up and Go (TUG), Berg Balance Scale (BBS), Activities-specific Balance Confidence (ABC), and Falls Efficacy Scale–International (FES-I). **Results:** After adjusting for baseline values, age, and BMI, the intervention group showed significantly greater improvements than the control group across all postural control outcomes. Notably, reductions in sway area, mean velocity, and sway path were observed under both eyes-open and eyes-closed conditions, with effect sizes ranging from moderate to very large (Cohen’s d = 0.57 to 1.61). For secondary outcomes, significant between-group differences were found in functional mobility (TUG), balance performance (BBS), and balance confidence (ABC), with moderate-to-large effect sizes (Cohen’s d = 0.53 to 0.73). However, no significant improvement was observed in fear of falling (FES-I), despite a small-to-moderate effect size. **Conclusions:** A supervised VR program significantly enhanced postural control, mobility, and task-specific balance confidence in frail older adults. These findings support the feasibility and efficacy of VR-based training as a scalable strategy for mitigating frailty-related mobility impairments.

## 1. Introduction

Frailty is a progressive geriatric syndrome characterized by reduced physiological reserve and diminished resilience to minor stressors, resulting in an elevated risk of adverse health outcomes such as falls, disability, and mortality [1,2]. In the context of global demographic transitions toward an aging population, the early detection and effective management of frailty are essential to prevent functional decline and maintain independence among older adults [3,4,5].

Several models have been developed to conceptualize and classify frailty, most notably the frailty phenotype and the cumulative deficit model, which underpins the Frailty Index [1,6,7]. However, substantial variability remains across frailty assessment tools. Studies have reported inconsistent prevalence estimates and differing associations with physical and psychological health outcomes [8]. Increasing attention has therefore been directed toward understanding not only the prevalence of frailty but also its functional consequences. Impairments in postural control, muscular strength, and mobility have been consistently linked to frailty status [9,10,11].

Although conventional exercise is a core component of frailty management, adherence among older adults is frequently limited. Contributing factors include low motivation, fear of falling, and restricted access to supervised programs, particularly in community settings [12,13]. In response, virtual reality-based interventions, including exergames and video game-guided exercise programs, have emerged as promising alternatives. Recent research has explored their potential to enhance physical and cognitive function among frail older adults [14,15].

Despite the growing interest in virtual reality applications, important questions remain. A central debate is whether exergames provide additional benefits compared to traditional exercise. Some researchers suggest that the motivational and cognitive engagement embedded in exergames may enhance physical performance and dual-task capabilities [16]. Others argue that when exercise intensity and volume are equivalent, exergames do not offer superior benefits and may even be less effective [14,17]. Furthermore, concerns have been raised regarding the appropriateness of virtual reality for frail individuals. While several studies report meaningful improvements, others highlight that cognitive impairments and physical limitations may hinder participation and necessitate individualized adaptations [15,18].

A recent randomized controlled trial by Chan et al. [19] investigated the feasibility and impact of a virtual reality intervention using the Nintendo Ring Fit Adventure™ among older adults with a history of falls. The study demonstrated significant improvements in balance based on functional assessments. However, its small sample size, limited range of outcome measures, and lack of long-term follow-up constrain the generalizability and sustainability of the findings. Notably, objective postural control metrics were not included, limiting the depth of evaluation. These methodological limitations point to the need for more rigorous studies employing both clinical and objective outcome measures to assess the efficacy of virtual reality interventions in frail populations.

In particular, objective posturographic metrics such as Center of Pressure (CoP) sway area, path length, and velocity are critical for providing a comprehensive understanding of balance-related improvements. In light of these gaps, the present study aimed to evaluate the effectiveness of a supervised virtual reality intervention using the Nintendo Ring Fit Plus™ on postural control, functional mobility, and balance confidence in community-dwelling frail older adults. Specifically, this study assessed whether a four-week structured virtual reality program could improve objective posturographic measures and clinical mobility outcomes, including the Timed Up and Go (TUG) test and the Berg Balance Scale (BBS). Additionally, this study examined changes in subjective perceptions of fall risk, using the Activities-specific Balance Confidence Scale (ABC) and the Falls Efficacy Scale–International (FES-I). We hypothesized that supervised virtual reality-based training would result in significant improvements in postural control and functional mobility among frail older adults compared to a control group.

## 2. Materials and Methods

### 2.1. Ethical Approval and Consent to Participate

The study protocol was reviewed and approved by the Ethical Advisory Committee at Umm Al-Qura University (Approval No. HAPO-02-K-012-2024-11-2337). The clinical trial was registered locally at Umm Al-Qura University in Saudi Arabia (UQUCT-2024-2-0043). Written informed consent was obtained from all participants after providing detailed information about the study’s objectives, procedures, potential benefits, and possible risks.

### 2.2. Participants and Study Setting

Participants were recruited from Mecca and surrounding communities using social media platforms and distributed newsletters. This was a non-randomized controlled study. Participants were allocated to groups based on scheduling availability and personal preference, ensuring feasibility and adherence to the supervised training protocol. All assessments and intervention sessions were conducted at Umm Al-Qura University. Outcome measures were recorded at baseline (week 0) and following the four-week intervention period (week 4).

### 2.3. Eligibility Criteria

Eligible participants were aged 65 years or older and identified as frail or prefrail according to the Fried Frailty Phenotype [20]. This index evaluates five components: unintentional weight loss, self-reported exhaustion, low physical activity, slow walking speed, and reduced grip strength. Individuals meeting one or two criteria were classified as prefrail, while those meeting three or more were classified as frail [7].

In this study, grip strength was measured using a hand-held dynamometer. Walking speed was assessed using the TUG test [21]. The remaining components, including unintentional weight loss, exhaustion, and physical activity level, were evaluated through structured self-report questions consistent with the original frailty phenotype protocol [22,23].

Participants were required to be able to walk independently, participate in standing exercises, and have normal or corrected vision and hearing. Exclusion criteria included contraindications to exercise, significant sensory or cognitive impairments, participation in concurrent physical interventions, or no history of falls within the past year.

### 2.4. Data Collection

Data were collected during a structured one-hour baseline session involving both clinical assessments and standardized interviews. The collected variables included age, sex, height, weight, body mass index (BMI), frailty classification, and fall history.

### 2.5. Outcome Measures

The primary outcomes were postural control metrics obtained through static posturography. These included CoP mean velocity, sway area (calculated as the 95 percent confidence ellipse), and sway path length. Secondary outcomes included the following:

- BBS: a clinical measure of static and dynamic balance;
- TUG test: a performance-based measure of functional mobility;
- ABC: a self-report measure of balance confidence during daily tasks;
- FES-I: a self-report measure of fear of falling.

### 2.6. CoP Data Acquisition and Procedures

This study followed general recommendations for stabilometry research [24,25]. Participants completed three 60 s quiet standing trials with their eyes open, followed by three additional trials with their eyes closed. Rest breaks were provided between trials as needed. The procedures for calculating CoP-related variables are detailed in Table S1.

### 2.7. Intervention

The intervention was provided over four weeks, with participants attending three sessions per week. Each session continued for 45–60 min and included structured warm-up, gameplay, and cooldown components. The intensity of the exercises was moderate and gradually increased based on participant tolerance and performance. The intervention consisted of supervised virtual reality exercise sessions using the Nintendo Ring Fit Adventure™ program, adapted for older adults. The specific game modules used in the training sessions are summarized in Table 1.

### 2.8. Sample Size Calculation

A priori power analysis was conducted using G*Power version 3.1.9.7 (Heinrich Heine University Düsseldorf, Düsseldorf, Germany) to estimate the required sample size. An analysis of covariance (ANCOVA) was chosen to adjust for baseline differences between groups in this non-randomized controlled study. A fixed effects ANCOVA model with two groups and three covariates (age, BMI, and pre-intervention score) was used. Assuming a moderate effect size (Cohen’s f = 0.45), an alpha level of 0.05, and a desired statistical power of 80 percent, the minimum required sample size was calculated to be 41 participants. The final sample of 51 participants provided an estimated power of 88.3 percent, indicating adequate power to detect moderate effects.

### 2.9. Statistical Analyses

All statistical analyses were performed using IBM SPSS Statistics version 27.0 (IBM Corp., Armonk, NY, USA). The distribution of continuous variables was evaluated using skewness and kurtosis, with values within ±1 considered indicative of normal distribution.

Descriptive statistics were presented as means and standard deviations for normally distributed variables, and as medians with interquartile ranges for variables not normally distributed. Independent samples *t*-tests were used to compare baseline characteristics between groups.

For the analysis of changes over time and between groups, ANCOVA models were applied, adjusting for baseline scores, age, and BMI, where model assumptions were met. Effect sizes were reported using Cohen’s d and interpreted as small (0.1), medium (0.4), or large (0.8) [26]. A *p*-value less than 0.05 was considered statistically significant.

## 3. Results

A total of 51 participants were enrolled in this study, with 28 allocated to the intervention group and 23 to the control group. The mean age of participants in the intervention group was 70.68 ± 4.03 years, whereas the control group was older, with a mean age of 74.52 ± 5.06 years. Gender distribution was relatively balanced between groups, with 13 females and 15 males in the intervention group and 11 females and 12 males in the control group. A summary of the demographic and clinical characteristics of participants is provided in Table 2.

Baseline comparisons revealed statistically significant differences in several demographic variables. Participants in the control group were significantly older than those in the intervention group (*p* = 0.006). Additionally, the intervention group demonstrated significantly higher values for height (*p* < 0.001), weight (*p* = 0.002), and BMI (*p* = 0.008). However, no significant between-group differences were observed in frailty levels as measured by the Fried Frailty Phenotype (*p* = 0.335). Similarly, gender distribution did not differ significantly between the two groups (*p* = 1.000).

Most study variables satisfied the assumptions of normality, as assessed by skewness and kurtosis values within ±1. Minor deviations were noted in a few self-reported variables, but these did not affect the overall validity of the parametric analyses.

### 3.1. Primary Outcomes

**Figure 1:**
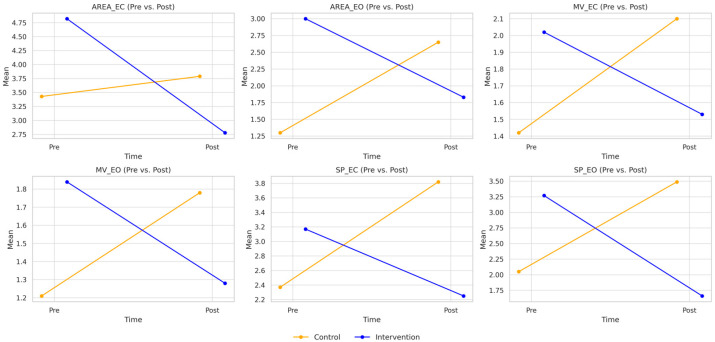
Changes in postural control metrics before and after the intervention in both groups. Blue lines represent the intervention group; orange lines represent the control group. Variables include sway area with eyes open (AREA_EO, cm^2^), sway area with eyes closed (AREA_EC, cm^2^), mean velocity with eyes open (MV_EO, cm/s), mean velocity with eyes closed (MV_EC, cm/s), sway path with eyes open (SP_EO, cm), and sway path with eyes closed (SP_EC, cm).

The analyses revealed significant post-intervention between-group differences across all postural sway parameters, consistently favoring the intervention group. Figure 1 presents the mean values and standard deviations for the six CoP metrics assessed under both eyes-open and eyes-closed conditions at baseline and post-intervention.

For sway area, significant post-intervention between-group differences were observed across both visual conditions (all, *p* < 0.05). In the eyes-open condition, the intervention group demonstrated a significantly (*p* < 0.001) lower sway area (1.83 cm^2^; 95% CI: 1.66–2.01) compared to the control group (2.65 cm^2^; 95% CI: 2.44–2.86). The mean difference was –0.82 cm^2^, corresponding to a large effect size (Cohen’s d = 0.90; 95% CI: 0.41–1.38). With eyes closed, the intervention group also exhibited a significantly (*p* = 0.037) lower sway area (2.78 cm^2^; 95% CI: 2.23–3.34) compared to the control group (3.79 cm^2^; 95% CI: 3.16–4.42). The mean difference was –1.01 cm^2^, with a corresponding moderate effect size (Cohen’s d = 0.57; 95% CI: 0.02–1.12).

For mean velocity, significant post-intervention between-group differences were also observed across both visual conditions (all, *p* < 0.001). In the eyes-open condition, the intervention group demonstrated a significantly (*p* < 0.001) lower mean velocity (1.28 cm/s; 95% CI: 1.17–1.39) compared to the control group (1.78 cm/s; 95% CI: 1.64–1.91). The corresponding Cohen’s d value was 1.04 (95% CI: 0.50–1.57). The mean difference was –0.49 cm/s, corresponding to a large effect size (Cohen’s d = 1.04; 95% CI: 0.50–1.57). With eyes closed, the intervention group showed a significantly (*p* < 0.001) lower mean velocity (1.53 cm/s; 95% CI: 1.40–1.67) compared to the control group (2.10 cm/s; 95% CI: 1.94–2.25). This difference yielded a mean of –0.56 cm/s, with a corresponding large effect size (Cohen’s d = 0.94; 95% CI: 0.41–1.47).

Similarly, significant post-intervention between-group differences were observed for sway path across both visual conditions (all, *p* < 0.001). In the eyes-open condition, the intervention group demonstrated a significantly (*p* < 0.001) lower sway path (1.66 cm; 95% CI: 1.41–1.90) compared to the control group (3.49 cm; 95% CI: 3.21–3.77). The mean difference was −1.84 cm, corresponding to a very large effect size (Cohen’s d = 1.61; 95% CI: 1.02–2.19). With eyes closed, the intervention group exhibited a significantly (*p* < 0.001) lower sway path (2.25 cm; 95% CI: 1.74–2.76) than the control group (3.82 cm; 95% CI: 3.24–4.39). This yielded a mean difference of −1.57 cm, with a corresponding large effect size (Cohen’s d = 0.87; 95% CI: 0.35–1.39).

### 3.2. Secondary Outcomes

**Figure 2:**
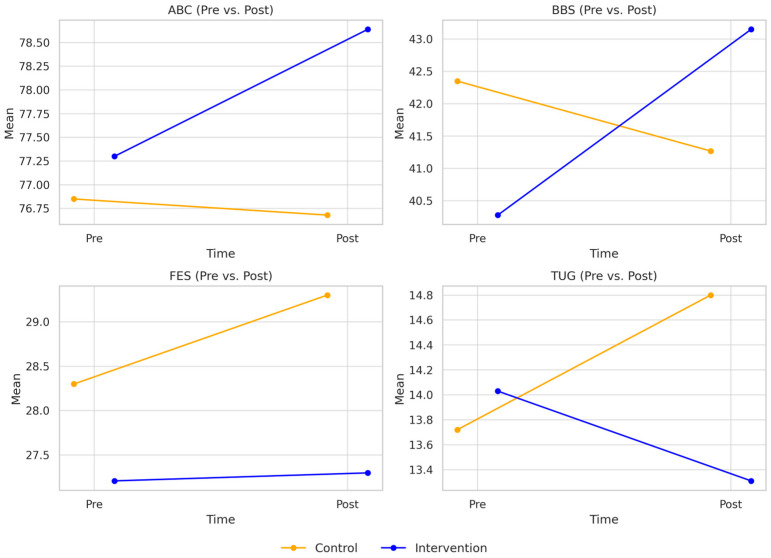
Pre- and post-intervention changes in clinical balance and mobility outcomes. TUG refers to the Timed Up and Go test in seconds, BBS is the Berg Balance Scale with a maximum score of 56, ABC is the Activities-specific Balance Confidence Scale scored out of 100, and FES-I is the Falls Efficacy Scale–International, scored out of 64.

Figure 2 presents the mean scores and standard deviations for the four secondary outcomes assessed at baseline and post-intervention in both groups. Significant post-intervention between-group differences were observed for several secondary outcomes. For the TUG test, a significant group effect was found (*p* = 0.006). The intervention group demonstrated a lower TUG time following the intervention (13.31 s; 95% CI: 12.66–13.95) compared to the control group (14.80 s; 95% CI: 14.08–15.53), yielding a mean difference of −1.49 s and a medium-to-large effect size (Cohen’s d = 0.73; 95% CI: 0.16–1.30). Similarly, for the BBS, a significant group effect was observed (*p* = 0.010), with the intervention group scoring higher (43.15; 95% CI: 42.30–44.00) than the control group (41.27; 95% CI: 40.31–42.23). The mean difference of 1.88 points corresponded to a moderate-to-large effect size (Cohen’s d = 0.69; 95% CI: 0.12–1.26). For the ABC scale, the intervention group also showed significantly (*p* = 0.033) greater confidence following the intervention (78.64; 95% CI: 77.54–79.75) compared to the control group (76.68; 95% CI: 75.43–77.92). The mean difference of 1.97 points corresponded to a moderate effect size (Cohen’s d = 0.53; 95% CI: 0.04–1.02). In contrast, no significant group effect was found for the FES-I (*p* = 0.136). Although the intervention group had a lower FES-I score following the intervention (27.30; 95% CI: 25.69–28.92) than the control group (29.30; 95% CI: 27.49–31.12), the mean difference of −2.00 points did not reach statistical significance. The corresponding effect size was small-to-moderate (Cohen’s d = 0.37; 95% CI: −0.14 to 0.89).

## 4. Discussion

This study investigated the effects of a supervised virtual reality training program using the Nintendo Ring Fit on postural control and functional mobility outcomes in a sample of older adults with frailty. The results demonstrated significant improvements in static postural control, as measured by CoP metrics, and in selected functional outcomes following the intervention. No comparable improvements were observed in the control group. These findings suggest that structured virtual reality training may offer a promising and engaging rehabilitation modality for frail older adults.

The significant group-by-time interaction effects across all CoP metrics reflect substantial improvements in static postural control among participants in the intervention group, with large effect sizes. In contrast, the control group showed either no improvement or a decline in performance. CoP-based parameters are considered highly sensitive indicators of subtle impairments in postural control and fall risk. They often detect neuromuscular adaptations before such changes become evident through clinical assessments [27]. The improvements observed in CoP outcomes align with the gains in clinical balance measures, such as the BBS and the TUG test. The concurrent improvements across both objective and clinical measures suggest that the intervention not only enhanced motor performance but also positively influenced the underlying mechanisms of postural control. This relationship is supported by previous research showing that CoP mean velocity and sway area correlate strongly with TUG and BBS scores in frail populations [28]. Furthermore, a recent systematic review and meta-analysis confirmed that virtual reality training significantly improves both static and dynamic CoP metrics compared to standard care [29]. The consistency between our findings and those reported in the literature reinforces the clinical relevance of CoP enhancements as predictors of reduced fall risk. Importantly, the inclusion of posturographic assessments in the current study contributes to the growing body of evidence linking virtual reality-based exercise to neurophysiological adaptations in balance control [30].

The improvements in TUG and BBS scores are consistent with previous investigations. For instance, Gandolfi et al. [31] reported that virtual reality-based training led to significant gains in postural control among older adults, emphasizing its potential role in reducing fall risk. Similarly, Carvajal-Parodi et al. [32] observed enhancements in both static and dynamic balance following exergaming interventions. However, it is important to recognize differences in study populations and intervention protocols. For example, Gandolfi et al. focused on individuals with cognitive impairment, which may limit the generalizability of their findings to cognitively intact older adults, such as those included in the current study.

While no significant group-by-time interaction was observed for the FES-I, the ABC scale demonstrated significant improvement in the intervention group, with a moderate effect size. This contrast highlights important differences in the conceptual focus and psychometric properties of the two measures [33]. Although both tools assess aspects of fall-related self-efficacy, the FES-I emphasizes fear of falling during daily activities and is more psychologically oriented. In contrast, the ABC scale captures confidence in performing balance-challenging tasks, such as walking in crowded environments or climbing stairs [34]. The significant improvement in ABC scores suggests that the virtual reality training enhanced participants’ confidence in managing complex, balance-demanding tasks, even if their general fear of falling remained unchanged. This pattern likely reflects the task-specific nature of the intervention, which involved dynamic movements, strength-based exercises, and postural challenges. These activities may have improved participants’ perceived capability to perform specific tasks, a construct more directly measured by the ABC scale.

Similar patterns have been reported in previous studies. For example, a study by [35] found that virtual reality training improved task-specific confidence in older adults during complex mobility tasks, with limited effects on generalized fear of falling. This supports the view that specific confidence gains may precede broader psychological improvements. Moreover, the improvements in ABC scores observed in the current study provide further evidence that virtual reality training can enhance task-specific self-efficacy, which is known to facilitate engagement and adherence to exercise programs in older adult populations [36]. These findings underscore the importance of including multiple, construct-valid self-report measures in fall prevention research to capture the multifaceted psychological responses to interventions.

The intervention in this study was delivered in a controlled, supervised laboratory setting, which ensured participant safety, adherence, and proper execution of the exercises. This supervised environment may have contributed to the observed training effects by providing structured progression and immediate feedback. Previous research by Ho et al. [37] similarly demonstrated that supervised virtual reality training resulted in greater adherence and better functional outcomes than unsupervised training in frail older adults. A 2023 systematic review also emphasized that, although exergaming consistently improves physical performance in older adults, variability in supervision, adherence, and program structure remains a limitation across studies [38].

Despite these encouraging findings, several limitations must be acknowledged. First, the intervention duration was limited to four weeks, and no follow-up assessments were conducted. Given the progressive nature of frailty and fall risk, long-term monitoring is essential to determine the sustainability of benefits. Second, although a control group was used, the absence of random allocation may have introduced selection bias and limits the ability to draw causal inferences. Future studies should adopt randomized controlled trial designs to enhance internal validity. Third, baseline differences between groups in variables such as age, height, weight, and BMI may have influenced both balance capacity and responsiveness to the intervention. While statistical adjustments were applied, the possibility of residual confounding cannot be ruled out. Fourth, although frailty is a multidimensional construct, this study did not include measures of psychosocial factors that could influence balance confidence. Inclusion of these domains may have provided a more comprehensive understanding of participant responses. Based on these limitations, future research should explore the feasibility and effectiveness of implementing this intervention in community or home-based settings. Larger, multicenter trials are recommended to enhance generalizability and statistical power. Cost-effectiveness analyses should also be included to assess the practicality of wider adoption. Additionally, incorporating cognitive dual-task components and real-time biomechanical feedback may improve the specificity and impact of virtual reality-based training programs.

## 5. Conclusions

This study showed that supervised virtual reality training is a feasible and effective intervention for improving postural control, functional mobility, and task-specific balance confidence in frail older adults. These findings support the integration of virtual reality-based training into rehabilitation programs for frail older adults. Future research should explore its long-term benefits, cost-effectiveness, and adaptability for home or community-based implementation.

## Figures and Tables

**Table 1:** Game modules used in the virtual reality exercise sessions.

Activity	Description	Category
Warrior II	A slow, controlled yoga posture reinforcing balance and hip strength. Safe and effective in improving anticipatory balance.	Balance
Wide Squat	A squat-based resistance exercise targeting lower limb and core strength.	Strength
Overhead Bend	A stability-focused activity that activates balance responses.	Balance
Knee Lift	A gentle knee-raising exercise that targets hip flexor strength and enhances functional mobility.	Strength/Mobility

Full protocol details are available in Table S2.

**Table 2:** Baseline characteristics of participants in the intervention and control groups.

Variable	Intervention (*n* = 28)	Control (*n* = 23)	*p*-Value
Age (years)	70.68 ± 4.03	74.52 ± 5.06	0.006
Sex (Male, %)	53.6%	52.2%	0.9
Height (cm)	172.50 ± 4.86	166.30 ± 4.40	<0.001
Weight (kg)	80.46 ± 9.75	70.35 ± 8.54	0.002
BMI (kg/m^2^)	27.39 ± 3.31	24.89 ± 2.18	0.008
Grip Strength (kg)	24.79 ± 2.85	25.08 ± 2.68	0.709
Fried score	3.36 ± 0.73	3.57 ± 0.59	0.335
Number of falls	3.32 ± 1.59	2.65 ± 1.03	0.088

## Data Availability

The original contributions presented in this study are included in the article; further inquiries can be directed to the corresponding author.

## Supplementary Materials

The following supporting information can be downloaded at https://www.mdpi.com/article/10.3390/jcm14155531/s1, Table S1: Describes the procedures for calculating CoP-related variables; Table S2: Provides full protocol details for the virtual reality training program. Refs. [39,40,41,42,43,44,45,46,47,48,49,50,51,52,53,54] are cited in Table S1.

## Abbreviations

The following abbreviations are used in this manuscript:

CoP	Center of Pressure
TUG	Timed Up and Go test
BBS	Berg Balance Scale
ABC	Activities-specific Balance Confidence Scale
FES-I	Falls Efficacy Scale—International
BMI	Body Mass Index
ANCOVA	Analysis of Covariance
