# Supplementary material for: Gaming Against Frailty: Effects of Virtual Reality-Based Training on Postural Control, Mobility, and Fear of Falling Among Frail Older Adults
Source: J Clin Med. 2025 Aug 6;14(15):5531. doi: 10.3390/jcm14155531 (PMC12347130; doi:10.3390/jcm14155531)
Supplement: Supplementary file 1 [file jcm-14-05531-s001.zip › Table S1 - Full calculation procedures for center of pressure (CoP) variables.pdf]

Table S1. Full calculation procedures for center of pressure (CoP) variables

The following calculation methods were adopted from Vieira et al. [1]:

Variable	Description
Sway path	"The length of CoP trajectory on the Base of Support (BoS)." As the CoP signal does not directly indicate changes in postural control, the sway path can be computed by summing the distance between successive CoP locations [2].
Mean velocity	"The total distance travelled by the CoP over time." It can be determined by dividing the sway path by the duration of the trial, and it demonstrates high reliability when a double-legged stance is employed (R = 0.84) [3].
Sway area or 95% confidence ellipse area	"The dispersion of the CP data through the calculus of the statokinesigram area" [4]. It was calculated by computing 95% confidence ellipse of COP AP and COP ML coordinates. In other terms, it includes 95% of the CoP samples within 60 seconds. The sway area was computed using principal component analysis to estimate its axes [5,6].

In this study, the COP signals were processed on a computer linked to the ADC, utilizing BioWare® software to acquire the COP traces. Throughout the trials, the COP position traces were recorded using a static force platform (Kistler®). A single force platform, with dimensions of 60 cm × 90 cm (42 cm × 70 cm between sensors), was used to collect all COP data during bipedal quiet standing. The data were sampled and recorded at a frequency of 100 Hz.

Raw COP data were processed using an automated code written in MATLAB (MATLAB, version R2019b, Usway area). The MATLAB scripts for calculating these parameters are provided in Table and were adapted from Prieto *et al.* [7] and Březina [8]. An increase in COP parameters indicates inadequate postural control, while a decrease signals better postural control. The variables analysed were sway area, mean velocity, and sway path in the AP and ML directions. All parameters were computed for each trial and then averaged.

MATLAB codes for COP parameters:

CoP Parameter	MATLAB Code
Sway path	sway path=sum(sqrt(CPap.^2+CPml.^2)).
Mean velocity	mean velocity=sum(sqrt(diff(CPap).^2+diff(CPml).^2))*freq/length(CPap).
Sway area or 95% confidence ellipse area	[vec,val]=eig(cov(CPap,CPml)); Area=pi*prod(2.4478*sqrt(svd(val))).

These parameters were selected because they are widely recognized as representative measures [9] and exhibit strong correlations with falls in older adults and are highly correlated with falls in older adults [10-13]. They also demonstrate high interrater and test-retest reliability, with ICC values ranging from 0.70 to 0.89 [14]. Mean velocity was utilized in this study as it has been shown to be the most discriminative COP parameter

for evaluating age group differences in postural control and fall risk [15,16]. Sway area was selected because prior research has demonstrated that combining mean velocity and sway area provides discriminative data between individuals with impaired postural control and those without impairments, whereas using sway area alone does not offer this level of differentiation [17].

References:

1. Vieira, M.F.; de Avelar, I.S.; Silva, M.S.; Soares, V.; Lobo da Costa, P.H. Effects of four days hiking on postural control. PLoS ONE 2015, 10, e0123214–e0123219.
2. Hufschmidt, A.; Dichgans, J.; Mauritz, K.H.; Hufschmidt, M. Some methods and parameters of body sway quantification and their neurological applications. Arch. Psychiatr. Nervenkr. 1980, 228, 135–150.
3. Le Clair, K.; Riach, C. Postural stability measures: what to measure and for how long. Clin. Biomech. 1996, 11, 176–178.
4. Duarte, M.; Freitas, S.M.S.F. Revision of posturography based on force plate for balance evaluation. Braz. J. Phys. Ther. 2010, 14, 183–192.
5. Kim, G.; Ferdjallah, M.; Harris, G.F. Fast differential analysis of center of pressure data in normal children and children with cerebral palsy. In Proceedings of the 25th Southern Biomedical Engineering Conference, Miami, FL, USA, 15–17 May 2009; McGoron, A.J., Li, C.Z., Lin, W.C., Eds.; IFMBE Proc. 2009, 24, 341–342.
6. Oliveira, L.F.; Simpson, D.M.; Nadal, J. Calculation of area of stabilometric signals using principal component analysis. Physiol. Meas. 1996, 17, 305–312.
7. Prieto, T.E.; Myklebust, J.B.; Hoffmann, R.G.; Lovett, E.G.; Myklebust, B.M. Measures of postural steadiness: differences between healthy young and elderly adults. IEEE Trans. Biomed. Eng. 1996, 43, 956–966.
8. Březina, T. Mechatronics 2017—Preface. Adv. Intell. Syst. Comput. 2018, 644.
9. Qiu, H.; Xiong, S. Center-of-pressure based postural sway measures: reliability and ability to distinguish between age, fear of falling and fall history. Appl. Ergon. 2015, 47, 37–44.
10. Maki, B.E.; Holliday, P.J.; Topper, A.K. A prospective study of postural balance and risk of falling in an ambulatory and independent elderly population. J. Gerontol. 1994, 49, M72–M84.
11. Stel, V.S.; Smit, J.H.; Pluijm, S.M.F.; Lips, P. Balance and mobility performance as treatable risk factors for recurrent falling in older persons. J. Clin. Epidemiol. 2003, 56, 659–668.
12. Tamburella, F.; Scivoletto, G.; Iosa, M.; Molinari, M. Reliability, validity, and effectiveness of center of pressure parameters in assessing stabilometric platform in subjects with incomplete spinal cord injury: A serial cross-sectional study. J. Neuroeng. Rehabil. 2014, 11, 86.

13. Topper, A.K.; Maki, B.E.; Holliday, P.J. Are activity-based assessments of balance and gait in the elderly predictive of risk of falling and/or type of fall? J. Am. Geriatr. Soc. 1993, 41, 479–487.
14. Swanenburg, J.; de Bruin, E.D.; Favero, K.; Uebelhart, D.; Mulder, T. The reliability of postural balance measures in single and dual tasking in elderly fallers and non-fallers. BMC Musculoskelet. Disord. 2008, 9, 162.
15. Moghadam, M.; Ashayeri, H.; Salavati, M.; Akhbari, B.; Ebrahimi, I.; Taghipour, M.; Mazaheri, M.; Negahban, H. Reliability of center of pressure measures of postural stability in healthy older adults: Effects of postural task difficulty and cognitive load. Gait Posture 2011, 33, 651–655.
16. Raymakers, J.A.; Samson, M.M.; Verhaar, H.J.J. The assessment of body sway and the choice of the stability parameter(s). Gait Posture 2005, 21, 48–58.
17. Riley, P.O. Phase plane analysis of stability in quiet standing. J. Rehabil. Res. Dev. 1995, 32, 227–235.
