# Supplementary material for: Gaming Against Frailty: Effects of Virtual Reality-Based Training on Postural Control, Mobility, and Fear of Falling Among Frail Older Adults
Source: J Clin Med. 2025 Aug 6;14(15):5531. doi: 10.3390/jcm14155531 (PMC12347130; doi:10.3390/jcm14155531)
Supplement: Supplementary file 1 [file jcm-14-05531-s001.zip › Table S2 - Full protocol of the virtual reality training program.pdf]

Table S2. Full protocol of the virtual reality training program

Overview

The virtual reality (VR) training program was delivered using the Nintendo™ **Ring Fit Adventure**™ platform, incorporating balance and strength-focused training tailored for older adults. The intervention aimed to enhance postural control, functional mobility, and reduce fall risk in frail older adults.

Setting and Supervision

- All sessions were conducted at Umm Al-Qura University in a designated lab space.
- A trained physical therapist supervised each session to ensure safety and provide assistance.

Frequency and Duration

- **Total duration**: 4 weeks
- **Frequency**: 3 sessions per week (total of 12 sessions)
- **Session duration**: ~30–40 minutes each, including warm-up and cool-down

Session Structure

Each session included the following components:

Component	Time	Description
**Warm-up**	5 minutes	Gentle dynamic stretches and low-intensity movements (e.g., marching in place)
**Main training**	25–30 minutes	VR-based exercises using Ring Fit Adventure™
**Cool-down**	5 minutes	Static stretching and breathing exercises

Game-Based Activities

Each participant completed a structured set of Ring Fit Adventure™ activities targeting specific domains as described in the manuscript. The following games were used consistently across sessions:

Progression Strategy

- **Week 1**: Low intensity and slower pace to promote adaptation, focusing on correct form and confidence.
- **Week 2–4**: Gradual increase in repetition and duration per activity, based on individual tolerance and therapist feedback.
- Game intensity was adjusted using the Ring Fit settings to match participant capacity while avoiding fatigue.

Safety Measures

- Each participant was screened before every session for fatigue, pain, or dizziness.
- Rest breaks were provided as needed.
- Participants were instructed to stop immediately and inform the therapist if any discomfort occurred.
- A stable support (e.g., chair or railing) was available nearby for safety during balance tasks.

Adherence and Compliance

- Attendance was recorded at each session.
- Compliance was monitored by the therapist and defined as completion of at least 10 out of 12 sessions (≥83%).

Individualization

- Initial session included familiarization with the Ring Fit system.
- Games were selected and adjusted based on participants' frailty level, physical function, and comfort.
- Verbal encouragement and positive reinforcement were used to maintain motivation and engagement.
